# Morphine-Driven m6A Epitranscriptomic Neuroadaptations in Primary Cortical Cultures

**DOI:** 10.1007/s12035-024-04219-z

**Published:** 2024-05-23

**Authors:** Konrad R. Dabrowski, Stephanie E. Daws

**Affiliations:** 1 Center for Substance Abuse Research, Temple University, Philadelphia, PA USA; 2 Department of Neural Sciences, Temple University, Philadelphia, PA USA; 3 Department of Biology, Temple University, Philadelphia, PA USA

**Keywords:** Morphine, Opioids, Neurons, N6-methyladenosine, m6A, RNA, Alkbh5

## Abstract

Opioid overdose is the leading cause of accidental death in the United States and remains a major public health concern, despite significant resources aimed at combating opioid misuse. Neurobiological research to elucidate molecular and cellular consequences of opioid exposure is required to define avenues to explore for reversal of opioid-induced neuroadaptations. Opioids impart well-documented regulation of the transcriptome and epigenetic modifications in the brain, but opioid-induced epitranscriptomic posttranscriptional regulation of RNA is vastly understudied. N6-methyladenosine (m6A) RNA methylation is significantly enriched in the brain and involved in learning, memory, and reward. m6A modifications have not been studied in opioid use disorder, despite being the most common RNA modification. We detected significant regulation of m6A-modifying enzymes in rat primary cortical cultures following morphine treatment, including AlkB Homolog 5 (*Alkbh5*). The m6a demethylase ALKBH5 functions as an m6A eraser, removing m6A modifications from mRNA. We hypothesized that chronic opioid treatment regulates m6A modifications through modulation of *Alkbh5* and profiled m6A modifications in primary cortical cultures following chronic morphine treatment and *Alkbh5* knock-down. We observed differential regulation of m6A modifications for a common set of transcripts following morphine or *Alkbh5* knock-down, and the two treatments elicited concordant m6A epitranscriptomic profiles, suggesting that a subset of morphine-driven m6A modifications may be mediated through downregulation of *Alkbh5* in cortical cultures. Gene Ontology terms of commonly regulated transcripts included serotonin secretion, synapse disassembly, neuron remodeling, and immune response. Thus, we conclude that morphine can drive epitranscriptomic changes, a subset of which may occur in an *Alkbh5*-dependent manner.

**Supplementary Information:**

The online version contains supplementary material available at 10.1007/s12035-024-04219-z.

## Introduction

Opioid use disorder (OUD) is one of the biggest public health challenges facing North America, with over 80,000 deaths attributed to opioids in 2022 alone [1]. The number of opioid overdoses nationally in the US has been increasing steadily over the last two decades, with a steepening increase due to the COVID-19 pandemic [2]. Predicted costs of OUD-related issues exceeds 78.5 billion US dollars each year [3]. Current pharmacological treatments for OUD, such as buprenorphine and methadone, target the mu-opioid receptor. However, the perseverant nature of OUD can be attributed to several long-term changes in neuronal structure and plasticity that are the result of transcriptomic [4] and epigenetic shifts [5]. OUD is a complicated, multifaceted disease that involves a broad range of molecular and circuit wide neuroadaptations that transcend the workings of the opioid receptor in controlling all aspects of the disease, including craving, relapse, tolerance, and other psychiatric pathologies. A comprehensive understanding of all the neuroadaptations and their impact on the disease development and progression is vital for devising successful therapeutic interventions for OUD. In the current study, we propose a new regulatory layer of opioid-induced neuroadaptations—epitranscriptomic regulation. The epitranscriptome is the collection of all chemical modifications that occur on RNA molecules that can influence their structure, stability, localization, and function in the cell [6]. Importantly, the impact of the epitranscriptome in opioid exposure has not yet been addressed. Here, we hypothesize that a specific type of epitranscriptomic modification called m6A is involved in the regulation of neural responses to opioids.

m6A, also known as N6-methyladenosine, is an abundant RNA modification that can be detected in brain cells [7, 8]. Studies have indicated that m6A is more abundant in the CNS than in any other tissue in the human body and m6A increases in overall abundance from the embryonic to adult brain [9]. RNA m6A modifications may impact a wide range of biological processes, including RNA splicing, stability, metabolism, translation, and localization [10–12]. In the nervous system, m6A RNA modifications have been demonstrated to modulate critical cellular and behavioral processing, such as adult neurogenesis, embryonic brain development, learning and memory, and axonal regeneration [13–16]. Enzymes that regulate m6A abundance on mRNA can be divided into three categories: m6A writers, m6A erasers, and m6A readers [10]. m6A is deposited co-transcriptionally by m6A writers that are part of the methyltransferase complex that consists of proteins such as methyltransferase 3 (METTL3), methyltransferase 14 (METTL14), and WT1 associated protein (WTAP). On the other hand, m6A can be removed from transcripts by RNA demethylases, commonly known as m6a erasers, such as fat mass and obesity associated protein (FTO) and alkB homolog 5 (ALKBH5) [10]. Finally, m6A can impact RNA stability, localization, and metabolism through the interaction with m6A readers, such as YTH N6-methyladenosine RNA-binding protein F1 (YTHDF1-3), YTH N6-methyladenosine RNA binding protein C1 (YTHDC1), or heterogeneous nuclear ribonucleoprotein A2/B1 (HNRNPA2B1) [17–19]. Very few studies have explored m6A modifications following exposure to drugs, and none have explored the consequences of opioid exposure on brain m6A modifications. However, the limited data available suggest that drug exposure results in m6A modification that may drive transcriptional patterns. For example, a multivariate genome-wide association meta-analysis identified FTO as one of the strongest associated genes with substance use disorder [20]. While FTO has been previously associated with increased body mass and obesity in humans [21–23], a recent study demonstrated that cocaine CPP results in decreased levels of *Fto* in the mouse hippocampus [24]. This data indicates that drug exposure may have the potential to regulate abundance of m6a modification through modulation of m6A modifying enzymes. Indeed, m6A epitranscriptomic shifts have been observed in the nucleus accumbens (NAc) of postmortem human subjects diagnosed with alcohol use disorder [25].

We sought to address these critical barriers by exploring the hypothesis that opioids may regulate gene expression through modification of m6A abundance in brain cells. The goal of the present study was to characterize m6A epitranscriptomic neuroadaptations induced by morphine exposure in primary cortical cultures and to identify m6A-modifying enzymes that are associated with opioid-induced m6A modification. We report that both *Alkbh5* and *Fto* are downregulated following chronic morphine treatment in primary cortical cultures. We performed an epitranscriptomic m6A microarray analysis on primary cortical cultures that underwent chronic morphine treatment or siRNA-mediated *Alkbh5* knock-down and observed overlap of a subset of morphine-induced m6A hypermethylation events with *Alkbh5* knock-down-induced m6A hypermethylation events. These data demonstrate that morphine exposure results in m6A epitranscriptomic changes in brain cells and identify *Alkbh5* as a putative regulator of opioid-induced m6A modifications.

## Materials and Methods

### Animals

Sprague Dawley rats were obtained from Charles River Laboratories (Wilmington, MA, USA). All procedures followed the National Institutes of Health’s Guide for the Care and Use of Laboratory Animals and were approved by Temple University’s Institutional Animal Care and Use Committee. Neuron enriched primary cortical cultures were prepared from P0 Sprague-Dawley rat pups that were rapidly decapitated. Rat brains were removed from the skull in HBSS (Gibco, Thermo Fisher Scientific, Frederick, MA, USA). Frontal cortex tissue was dissected under a dissecting microscope, as previously described [26], dissociated in primary neuron media, which consisted of DMEM with L-glutamine (Gibco, Thermo Fisher Scientific) with 2% B27 (Gibco, Thermo Fisher Scientific), 25mM HEPES (Sigma-Aldrich, St. Louis, MO, USA), and 7.8 g/L dextrose (Sigma-Aldrich), and plated onto 6-well plates (Corning, Corning, NY, USA) coated with poly-d-lysine (Gibco, Thermo Fisher Scientific). Neural cells were plated at a density of 500,000 cells per well and cultured in media for 7 days in vitro (div). Cells that underwent chronic morphine treatment were incubated with 20 μM heroin solution in media for 72 h starting on div 7. Cells were harvested for molecular analysis on div 10 by washing twice with ice-cold 1X phospho-buffered solution (PBS), followed by application of 700 μL of QIAzol lysis reagent (Qiagen, Hilden, Germany). Cell lysates were removed from plates using a cell scraper and lysates frozen at − 80 °C until RNA extraction.

### RNA Extraction

Total RNA was extracted from primary neural cultures using the miRNeasy Mini kit (Qiagen, Hilden, Germany), according to manufacturer’s instructions. RNA was suspended in RNase free water, and the concentration was measured using the Qubit HS Assay Kit (Life Technologies Corporation of Thermo Fisher Scientific, Frederick, MA, USA).

### Quantitative Polymerase Chain Reaction (qPCR)

A 500 ng of RNA was used as a template for cDNA synthesis. cDNA synthesis was performed as previously described [27]. Reverse transcription was performed in a 20 μL reaction mix containing 200 units (U) of Maxima Reverse Transcriptase (Thermo Fisher Scientific), 20 U of RiboLock (Thermo Fisher Scientific), 1 μL of 100 μM random hexamer primers (Thermo Fisher Scientific), 1 μL of 10 mM dNTP mix (Thermo Fisher Scientific), and 4 μl 5X RT Maxima RT buffer (Thermo Fisher Scientific). The reaction was incubated for 10 min at 25 °C, 30 min at 50 °C, and inactivated for 5 min at 85 °C in a miniAmp Thermal Cycler (Thermo Scientific). The resulting cDNA was diluted 20x and used as a template for qPCR reactions. The qPCR analysis was performed using IDT PrimeTime Gene Expression Mastermix, IDT PrimeTime qPCR Probe Assays (Integrated DNA Technologies, IDT, Coralville, Iowa), and a Quantstudio 3 qPCR machine (Thermo Fisher Scientific). The following endogenous controls were used: beta actin (*Actb*) and glyceraldehyde 3-phosphate dehydrogenase (*Gapdh*). A full list of all primers used in the study is located in the Supplemental Table 1. The 2^−ΔΔCt^ method was used to calculate the expression levels of measured transcripts [28].

### In Vitro siRNA Transfection

In vitro siRNA transfection of primary cortical cultures was performed using RNAiMax (Thermo Fisher Scientific) according to the manufacturer’s protocol, with minor modifications. Cells were transfected with pools of 4 siRNAs: either ON-TARGETplus Non-targeting pool (scrambled siRNA) or ON-TARGETplus Rat *Alkbh5* siRNA pool (Horizon Discovery Biosciences Limited, Cambridge, UK) (Supplemental Table 2). In short, 5 μL of the 20 μM siRNA stock was mixed with 250 μL of reduced serum medium OPTI-MEM (Gibco, Thermo Fisher Scientific). A 3 μL of RNAiMax transfection reagent was mixed with 250 μL of OPTI-MEM. Both reaction mixes were then combined and incubated at room temperature for 10 min before being added dropwise to primary cortical cultures. After 6 h, 50% of the media was replaced with prewarmed fresh primary neuron media. Cells were transfected on div 7 and harvested after 72 h on div 10.

### m6A Epitranscriptomic Microarray Analysis

Evaluation of m6A hyper- and hypomethylation in RNA samples from primary cortical cultures was performed by Arraystar Inc. (Rockville, MD, USA), as previously described [29]. For the epitranscriptomic microarray, we used 3.8 μg of total RNA from three replicates per group (vehicle, morphine, scrambled siRNA or anti-*Alkbh5* siRNA). Each replicate was a result of pooling 950 ng of RNA from 4 wells of a 6 well plate. In short, total RNA and m6A spike-in control mixture were added to 300 μL of 1x immunoprecipitation buffer containing 50 mM Tris-HCl, pH 7.4, 150 mM NaCl, 0.1% NP40, 40 U/μL RNase Inhibitor, and 2 μg of anti-m6A rabbit polyclonal antibody (Synaptic Systems, Göttingen, Germany). The reaction mixture was incubated for 2 h at 4 °C with head-over-tail rotation. Next, 20 μL per sample of Dynabeads™ M-280 Sheep Anti-Rabbit IgG suspension was blocked with 0.5% BSA for 2 h at 4 °C, washed three times in 1× immunoprecipitation buffer and resuspended in the previously prepared RNA-antibody mixture. The resulting mixture was incubated for 2 h at 4 °C with head-over-tail rotation. Beads were then washed once with 500 μL of 1× immunoprecipitation buffer and twice with 500 μL of wash buffer containing 50 mM Tris-HCl, pH7.4, 50 mM NaCl, 0.1% NP40, and 40 U/μL RNase inhibitor. RNA was eluted from the beads with 200 μL of elution buffer containing 10 mM Tris-HCl, pH7.4, 1 mM EDTA, 0.05% SDS, 40 U Proteinase K, 1 μL RNase inhibitor for 1 h at 50 °C. Acid phenol-chloroform with ethanol precipitation was used as the RNA extraction method. The immunoprecipitation RNA (IP) and supernatant RNA (Sup) were added with equal amount of m6A spike-in calibration control. They were separately amplified, and Sup RNA was labeled with Cy3, while IP RNA was labeled with Cy5 using the Arraystar Super RNA Labeling Kit. NanoDrop ND-1000 was used to measure the concentration and specific activity (pmol dye/μg cRNA) of samples. 2.5 μg of labeled IP (Cy5) and Sup (Cy3) cRNA were mixed and fragmented by the addition of 5 μL of 10× blocking agent and 1 μL of fragmentation buffer. The mixtures were incubated for 30 min at 60 °C and added to 25 μL of 2x hybridization buffer. A 50 μL of the hybridization reaction mixture was added to the m6A-mRNA&lncRNA Epitranscriptomic Microarray slide. The microarray slides were incubated for 17 h at 65 °C in an Agilent Hybridization Oven. Following hybridization, trays were washed, fixed, and scanned with an Agilent Scanner G2505C. Acquired array images were analyzed using Agilent Feature Extraction software (version 11.0.1.1). Raw intensities of the Cy5-labeled IP and Cy3-labeled Sup were normalized to the average of log2 intensities of spike-in RNA. m6A quantity was calculated using the IP normalized intensities. Differential methylation ≥ 1.5 or 2.0 fold change with a *p*-value ≤ 0.05 was considered statistically significant. Total mRNA expression changes were calculated using IP Cy5 normalized intensity values and Sup Cy3 normalized intensity values from the m6A epitranscriptomic microarray analysis. Total mRNA expression is equal to the sum of IP signal and Sup signal. Significance for transcript level changes was assumed at *p*-value ≤ 0.05 and fold change ≥ 1.5 or 2.0. Lists of significantly hyper- or hypomethylated genes were used as the input for Gene Ontology analysis using the functional annotation tool from the Database for Annotation, Visualization and Integrated Discovery (DAVID version 6.8 ; https://david.ncifcrf.gov). “GOTERM_BP_DIRECT,” “GOTERM_CC_DIRECT,” “GOTERM_MF_DIRECT” Gene Ontology terms with corresponding unadjusted *p*-values and unadjusted *p*-values for differentially m6A methylated transcripts were obtained to generate the bubble plots using the R package ggplot2 (version 3.4.2; Villanueva & Chen [30]). For identification of transcripts localized to the synapse, the SynGO (https://www.syngoportal.org) [31] was utilized. Input for SynGO was gene names of differentially methylated transcripts following morphine treatment (568 genes) or Alkbh5 knock-down (2865 genes). Synaptic mapping was also performed by comparing the differentially m6A methylated mRNA transcripts with the proteomic analysis of synaptosomes sorted via fluorescent-activated synaptosome sorting (FASS) [32]. Significance of the overlap was evaluated via Fisher’s exact test. Rank-rank hypergeometric analysis (RRHO) was performed to evaluate the similarity between transcriptomic signatures of two distinct experimental treatments (e.g., m6A *Alkbh5* knock-down and m6A morphine treatment). The RRHO2 package optimized by the Li Shen lab at Icahn School of Medicine at Mount Sinai (https://github.com/shenlab-sinai/RRHO2) was used to generate the stratified RRHO plots. GeneOverlap package in R Bioconductor, version 1.36.0 [33] was used to perform the Fisher’s exact test to compare the gene lists for each condition and evaluate the significance of the overlap. Drug gene interaction was performed on the differentially m6A methylated genes following the chronic morphine treatment or *Alkbh5* knock-down using the Drug-Gene Interaction Database (https://www.dgidb.org; Freshour et al. [34]). The list of identified drug-gene interactions was subsetted for opioids using the following terms: apomorphine, apomorphine hydrochloride, apomorphine hydrochloride hemihydrate, codeine, diacetylmorphine, fentanyl, heroin, hydrocodone, hydromorphone, levorphanol, methadone, morphine, oxycodone, oxymorphone, tramadol, propoxyphene, R-N-propylnorapomorphine, and (S)apomorphine.

### Statistical Analysis

All data are presented as mean ± standard error of the mean (SEM). D’Agostino normality tests were performed on all datasets. Unpaired student’s *t*-tests were used to analyze differences between two groups with normal distributions. Nonparametric Mann-Whitney tests were performed to compare differences between two groups without a normal distribution.

A *p*-value of less than 0.05 (*p* < 0.05) was considered statistically significant. Statistical analyses of qPCR data were performed on ddCT (delta-delta Ct) values prior to log transformation of fold change. The ROUT method (*Q* = 1%) in GraphPad was employed for outlier detection. In addition, samples were excluded from the RT-qPCR analysis in case of no amplification, as defined by a Ct value ≥ 35. In case of the epitranscriptomic m6A microarray, statistical significance was estimated using an unpaired Student *t*-test (significance defined as: *p*-value ≤ 0.05 and fold change ≥ 1.5 or 2.0). Fisher’s exact test was used to evaluate the significance of the overlap between any two lists of significantly m6A methylated or differentially expressed transcripts with *p*-value ≤ 0.05 considered as significant overlap. Statistical analyses of the qPCR data were done using the GraphPad software package (Prism version 9; GraphPad, San Diego, California, USA). Analysis of the m6A epitranscriptomic microarray was performed using Agilent Feature Extraction software (Agilent Technologies, Santa Clara, CA, USA). All other statistical analyses were performed using R version 4.3.1, unless specified otherwise.

## Results

### Chronic Morphine Exposure Leads to Decreased Levels of *Fto* and *Alkbh5* in Cortical Cultures

**Fig. 1:**
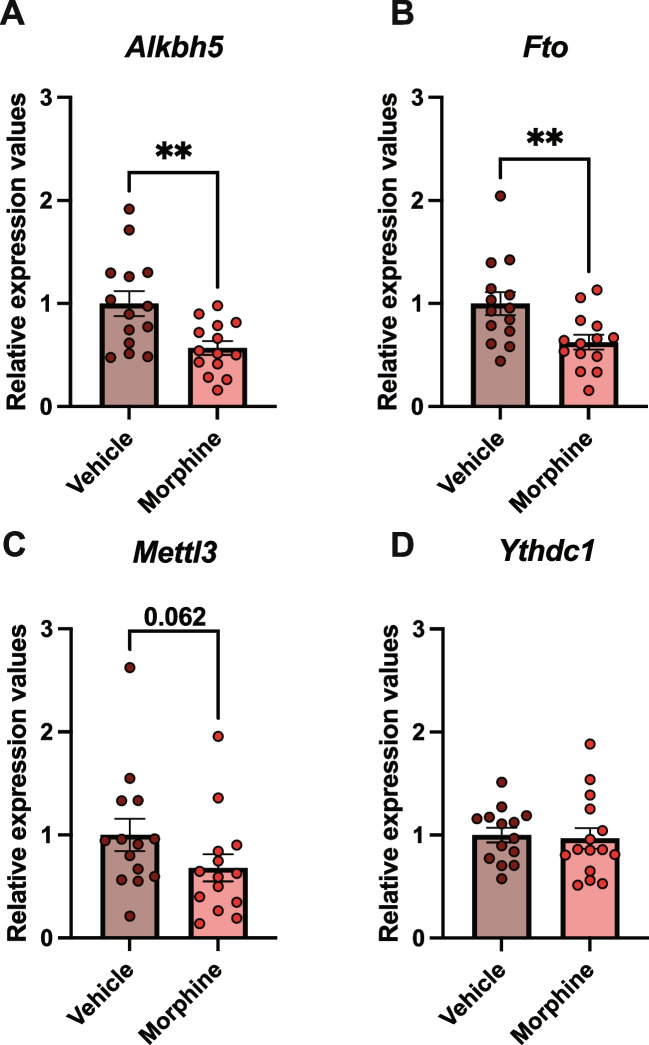
Chronic morphine treatment downregulates mRNA expression of m6 demethylases in primary cortical cultures. mRNA expression of **A** *Alkbh5*, **B** *Fto*, **C** *Mettl3*, and **D** *Ythdc1* in primary cortical cultures following chronic morphine treatment, obtained by qPCR. *N* = 14–15. Mean ± SEM. *p < 0.05, **p < 0.01

To determine the consequences of opioids on the epitranscriptome, we first interrogated the impact of opioid exposure on expression of enzymes that add (writers) or remove (erasers) m6a modifications to RNA. To evaluate whether opioid exposure can modulate the levels of m6A regulators in the nervous system, we isolated primary cortical cultures from P0 Sprague Dawley rats and exposed them to 20 μM morphine for 72 h between div7 and div10. Cultures from the frontal cortex were utilized for this study because the prefrontal cortex is a critical component of the brain’s reward system and modulates drug-induced molecular and behavioral neuroadaptations [35–37]. The mRNA expression of *Alkbh5*, *Fto*, *Mettl3*, and *Ythdc1* was measured with qPCR in chronic morphine-treated primary cortical cultures (Fig. 1). We observed a significant downregulation of *Alkbh5* (unpaired *t*-test: *t*(26) = 3.125, *p*-value = 0.0043; Fig. 1A) and *Fto* (unpaired *t*-test: *t*(26) = 2.837, *p*-value = 0.0087; Fig. 1B) compared to vehicle-treated cells. *Mettl3* also displayed a pattern of downregulation but did not reach statistical significance (Mann-Whitney test: *U* = 57, *p*-value = 0.062; Fig. 1C). Levels of *Ythdc1* were stable between conditions (Fig. 1D). These results demonstrate that chronic morphine treatment induces a downregulation of the m6A erasers *Alkbh5* and *Fto* in primary cortical cultures.

### Chronic Morphine Treatment Induces Significant Shifts in the m6A Epitranscriptomic Profile of Primary Cortical Cultures

**Fig. 2:**
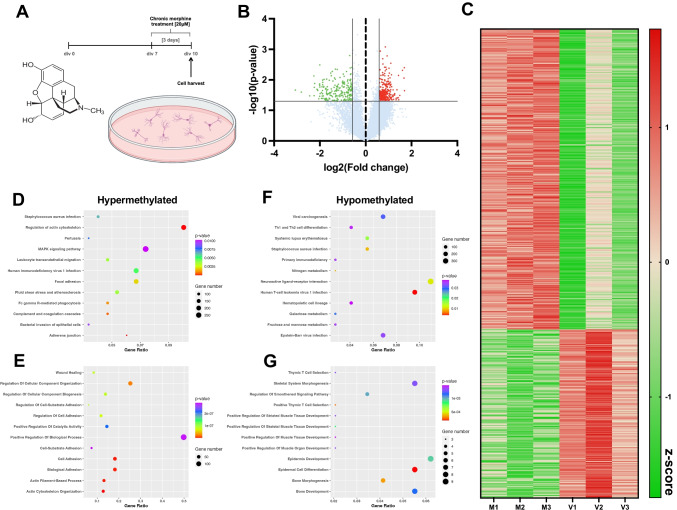
Chronic morphine treatment induces significant shifts in the m6A epitranscriptomic profile of primary cortical cultures. **A** Experimental overview. **B** Volcano plot depicting differential m6A methylation of transcripts in primary cortical cultures in response to chronic morphine treatment (*N* = 3). **C** Heatmap showing significantly regulated m6A methylation of transcripts between vehicle treated (V1, V2, and V3) and morphine treated (M1, M2, and M3) primary cortical cultures. Data expressed as a z-score of normalized expression data. **D**–**G** Bubble plot visualizations of KEGG pathway (**D**, **F**) and Gene Ontology (**E**, **G**) analysis for biological processes for hypermethylated (**D**, **E**) or hypomethylated (**F**, **G**) transcripts

The regulation of m6a erasers by morphine in cortical cultures suggests that morphine has the potential to shift m6A epitranscriptomic profiles. To gain more insight into the possible impact of differential regulation of m6A erasers following morphine exposure, we performed an m6A epitranscriptomic microarray on primary cortical cultures that underwent chronic morphine treatment (Fig. 2A). A key benefit of the epitranscriptomic microarray technology is the quantification of m6a methylation for each mRNA and noncoding RNA transcript, as well as detection of differential total mRNA expression for a given transcript. We detected 568 differentially methylated mRNA transcripts following chronic morphine treatment, with 364 hypermethylated and 204 hypomethylated (Fig. 2B, C and Supplemental Excel Table SE1). The m6A hypermethylated transcripts were associated through KEGG pathway analysis with terms that included MAPK signaling pathway, focal adhesion, adherens junctions, regulation of actin cytoskeleton, and several inflammation-related terms such as HIV-1 infection, bacterial invasion of epithelial cells, and *Staphylococcus aureus* infection (Fig. 2D). Gene Ontology of biological processes detected enrichment of hyper-methylated genes in terms associated with adhesion and actin cytoskeleton: actin cytoskeleton organization, actin-filament-based process, and cell adhesion (Fig. 2E). Hypomethylated genes were associated through KEGG pathway analysis with metabolism pathways (nitrogen, galactose, fructose, and mannose metabolism), and interestingly, with infection and immunity-related terms (e.g., primary immunodeficiency, *Staphylococcus aureus* infection, and Epstein-Barr virus infection) (Fig. 2F). Gene Ontology showed enrichment of hypomethylated genes including terms associated with cell development and morphogenesis (Fig. 2G). These data demonstrate that morphine exposure regulates m6a modification on mRNAs that code for proteins in many critical cellular processes related to cell signaling cascades, cytostructural components and inflammation.

### *Alkbh5* Knock-Down Leads to Hypermethylation of Transcripts Associated with Immunity Related Terms

**Fig. 3:**
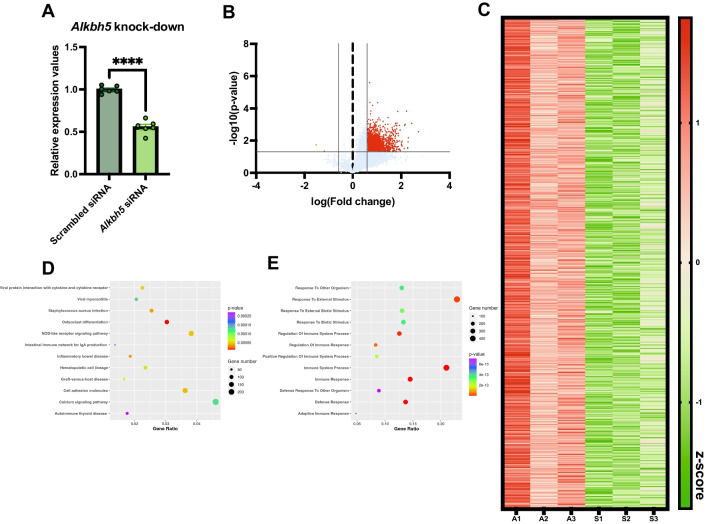
*Alkbh5* knock-down results in global hypermethylation of transcripts in primary cortical cultures. **A** qPCR validation of siRNA-based *Alkbh5* knock-down in primary cortical cultures (*N* = 6). **B** Volcano plot showing differential m6A methylation of transcripts in primary cortical cultures following *Alkbh5* knock-down. **C** Heatmap depicting significantly regulated m6A methylation of transcripts between scrambled siRNA (S1, S2, and S3) and *Alkbh5*-targetting siRNA (A1, A2, and A3) transfected primary cortical cultures. Data expressed as a *z*-score of normalized expression data. **D**, **E** Bubble plot visualization of the KEGG pathway (**D**) or Gene Ontology (**E**) analysis of hypermethylated transcripts. Mean ± SEM. ****p < 0.0001

Since we observed a downregulation of *Alkbh5* in primary cortical cultures in response to morphine, we next sought to identify mRNA transcripts that are regulated through ALKBH5-driven hypomethylation. To accomplish this, we performed siRNA knock-down of *Alkbh5* in primary cortical cultures, followed by epitranscriptomic array. We observed a 44% downregulation of *Alkbh5* following transfection with an *Alkbh5* siRNA, compared to cells transfected with a non-targeting scrambled control siRNA (Fig. 3A). As expected, knock-down of *Alkbh5*, an m6A eraser, led to global m6A hypermethylation (Fig. 3B). We detected 2865 differentially methylated mRNA transcripts following *Alkbh5* knock-down, of which > 99% were hypermethylated (Fig. 3B, C and Supplemental Excel Table SE2). Hypermethylated genes following *Alkbh5* knock-down were associated through KEGG pathway analysis with immunity- and infection-related terms (e.g., *Staphylococcus aureus* infection, inflammatory bowel disease, and viral myocarditis), as well as genes involved in cell adhesion, NOD-like receptor signaling and calcium signaling pathways (Fig. 3D). Gene Ontology associated the hypermethylated genes with a number of terms related to the immune response (e.g., immune response, immune system process, and regulation of the immune system process) (Fig. 3E).

Because the pathway and Gene Ontology analysis demonstrated that the differentially m6A-methylated transcripts in response to morphine were related to the organization of the cytoskeleton and cell adhesion (Fig. 2D–G), we examined the transcripts that were differentially m6A-methylated in our dataset in the context of synaptic location and function in the SynGO database. The analysis with SynGo resulted in sunburst plots mapping differentially methylated genes to different synaptic localizations (Supplemental Figure 1A-B). No significant enrichment for the synapse was detected; however, out of 568 differentially m6A-methylated transcripts following morphine treatment, 35 genes mapped to synaptic location (presynapse – 14, postsynapse - 22) (Supplemental Figure 1A and Supplemental Excel Table SE3). In addition, we performed the SynGO analysis on the differentially methylated transcripts following *Alkbh5* knock-down. Out of 2614 differentially m6A-methylated genes following *Alkbh5* knock-down, 137 were annotated to a synaptic location with 60 mapped to presynapse and 83 mapped to postsynapse (Supplemental Figure 1B and Supplemental Excel Table SE4). A recent study has reported 1800 unique synapse-type-enriched proteins via fluorescent-activated synaptosome sorting followed by proteomic analysis expanding our understanding of the proteins located at the synapse [32]. We compared the differentially m6A methylated genes induced by morphine and *Alkbh5* knock-down, and the aforementioned proteomic data (Supplemental Figure 1C-D). We observed a significant overlap of 51 genes between morphine-methylated genes and synapse-associated datasets (Fisher exact test: *p*-value = 0.032). The overlap between *Alkbh5* KD methylated genes and synapse-associated datasets, however, was not significant, but exhibited a trend towards significance (Fisher exact test: *p*-value = 0.051). These results support previous studies pointing to synaptic pathology in OUD [35]. Among the morphine differentially m6A-methylated transcripts, we observed genes encoding voltage-gated calcium channels (*Cacng3*) and a metabotropic glutamate receptor (*Grm7*). Multiple genes encode proteins important for cell adhesion (*Adgrl3*, *Cdh6*, *Cntn6*, *Nrcam*, *Pcdh15*, *Taok2*), synaptic transmission (*Clstn2*, *Cyfip1*, *Cyfip2*, *Stxbp1*), and endocytosis (*Dnm3*, *Flot1*, *Itsn1*, *Nrp2*, *Synj2*, *Syt11*) (Supplemental Excel Table SE3). Thus, a subset of differentially m6A-methylated transcripts regulated following morphine treatment or *Alkbh5* knock-down have synaptic localization.

### Morphine Exposure and *Alkbh5* Knock-Down Result in Concordant Epitranscriptomic Changes in Primary Cortical Neurons

**Fig. 4:**
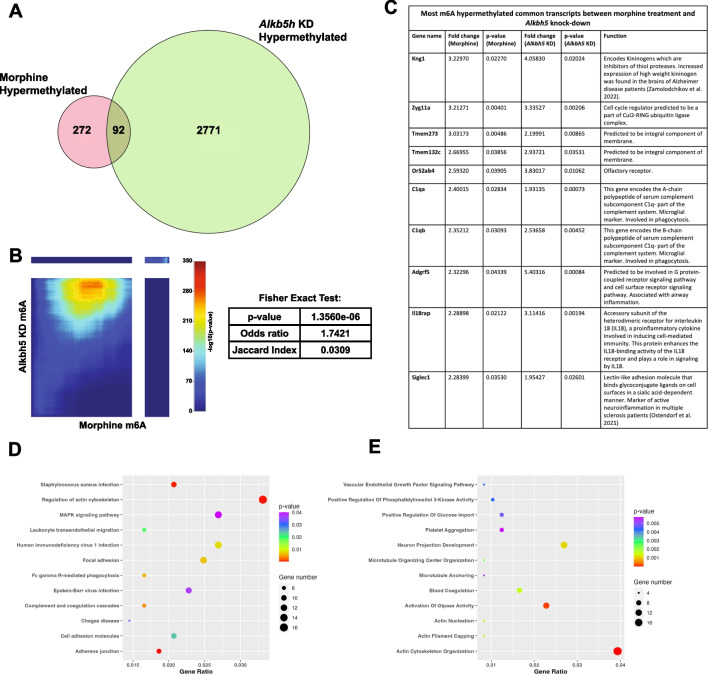
A subset of hypermethylated genes is commonly regulated by chronic morphine treatment and by *Alkbh5* knock-down in primary cortical cultures. **A** Venn diagram depicting overlap between the hypermethylated transcripts following chronic morphine treatment or *Alkbh5* knock-down in primary cortical cultures. **B** RRHO plot and Fisher exact test showing coordinated gene expression between *Alkbh5* siRNA knock-down and chronic morphine treatment conditions. In the lower left quadrant are genes that are upregulated in both datasets, while in the upper right quadrant are genes that are downregulated in both datasets. **C** A table of the top 10 transcripts that are commonly hypermethylated by morphine and *Alkbh5* knock-down. **D**, **E** Bubble plot visualizations of the KEGG pathway (**D**) and Gene Ontology (**E**) analysis for biological processes of the transcripts that are commonly hypermethylated by morphine and *Alkbh5* knock down

To assess whether morphine-induced m6A epitranscriptomic changes could be, in part, attributed to *Alkbh5* downregulation, we examined overlap between morphine hypermethylated mRNA transcripts and *Alkbh5* knock-down-induced epitranscriptomic changes. Ninety-two transcripts were commonly regulated between the two conditions (Fig. 4A and Supplemental Excel Table SE5). We next evaluated the similarity of morphine- and *Alkbh5* knock-down induced m6A epitranscriptomic profiles by performing a RRHO analysis on the two datasets. RRHO analysis is a statistical method used to compare two ranked gene lists to identify similarities between them and determine whether there are shared biological processes or pathways between the conditions that are being compared [38]. RRHO analysis accounts for directionality of change and p-value for all measured transcripts. We observed coordinated gene expression between morphine treatment in the two groups (Fisher’s exact test: *p* = 1.356 * 10^−06^), with an odds ratio of 1.7421 suggesting an association between the datasets (Fig. 4B and Supplemental Excel Table SE6). The top 10 genes that were most hypermethylated by both morphine treatment and *Alkbh5* knock-down are listed in Fig. 4C. The genes that were commonly hypermethylated by both morphine and *Alkbh5* knock-down were associated through KEGG pathway analysis with MAPK signaling pathway, adhesion (e.g., focal adhesion, adherens junctions, and leukocyte transendothelial migration), regulation of actin cytoskeleton, and inflammation-related terms (e.g., *Staphylococcus aureus* infection, HIV-1 infection, and Epstein-Barr virus infection) (Fig. 4D). These genes were associated through Gene Ontology analysis with the following biological processes: neuron projection development, actin cytoskeleton organization, and activation of Gtpase activity (Fig. 4E). These data demonstrate that morphine-induced hypermethylation of mRNA mirrors the epitranscriptomic signature of *Alkbh5* knock-down.

### A Subset of Differential m6A Methylation Events is Accompanied by Corresponding Transcriptomic Changes

**Fig. 5:**
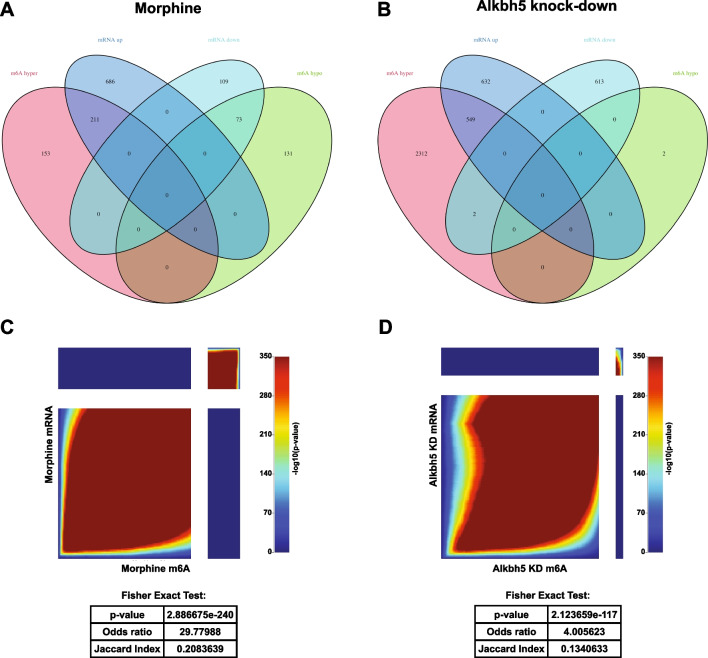
Differential m6A methylation is accompanied by altered RNA expression after chronic morphine treatment or *Alkbh5* knock-down. Venn diagrams depicting overlap between m6A hypermethylated (m6A hyper), m6A hypomethylated (m6A hypo), mRNA upregulated (mRNA up), and mRNA downregulated (mRNA down) transcripts following morphine treatment (**A**) or *Alkbh5* knock-down (**B**). **C**, **D** RRHO plots and Fisher exact tests showing coordinated gene expression between morphine induced m6A and mRNA changes (**C**), or *Alkbh5* knock-down induced m6A and mRNA changes (**D**)

One of the functions of m6A modifications is regulation of mRNA stability and metabolism. Therefore, we examined whether the m6A methylation changes following chronic morphine or *Alkbh5* knock-down are accompanied by corresponding changes to the mRNA transcript levels. Total mRNA changes were calculated from the microarray analysis following chronic morphine treatment or *Alkbh5* knock-down and compared to observed m6A epitranscriptomic changes for the same mRNA transcripts. Fifty percent of morphine differentially methylated mRNAs had a corresponding change in total transcript levels, including 211 hypermethylated that were upregulated and 73 hypomethylated genes that were downregulated (Fig. 5A). In cells with *Alkbh5* knock-down, 549 hypermethylated transcripts were also upregulated at the mRNA level (Fig. 5B). To further support the hypothesis that m6a methylation status is associated with mRNA transcript levels, we evaluated the overlap of m6A mRNA methylation and total mRNA expression datasets for both morphine treatment and *Alkbh5* knock-down (Fig. 5C, D). RRHO graphs show strong concordance, and association between transcripts with methylation changes and mRNA total expression changes (Fisher’s exact test, morphine: *p* = 2.887 * 10^−240^; *Alkbh5* knock-down: *p* = 2.124 * 10^−117^), suggesting that m6A-driven regulation of mRNA expression levels may be a significant mode of regulation in neural cells (Fig. 5C, D and Supplemental Excel Table SE6).

**Fig. 6:**
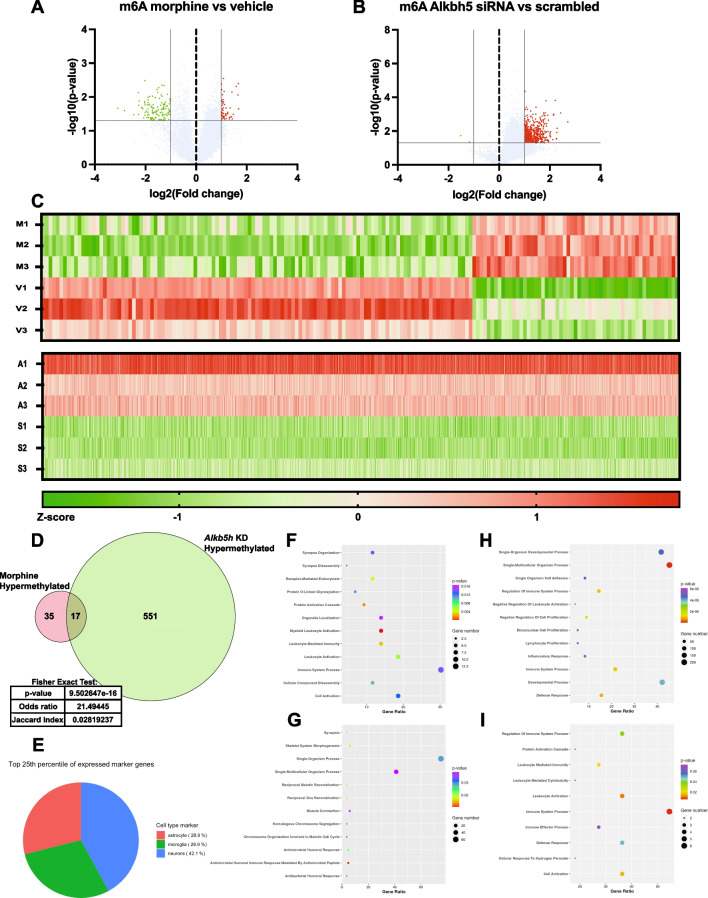
Significant overlap of m6A methylated transcripts between morphine treatment and *Alkbh5* knockdown is maintained following implementation of > 2 fold change criteria. Volcano plots depicting differential m6A methylation of transcripts in primary cortical cultures in response to chronic morphine treatment (**A**) or *Alkbh5* knock-down (**B**) using a more stringent fold change cutoff of > 2. **C** Heatmaps representing the significantly regulated m6A methylation of transcripts in primary cortical cultures between vehicle (V1, V2, and V3) and morphine (M1, M2, and M3) as well as between scrambled siRNA (S1, S2, and S3) and *Alkbh5* siRNA (A1, A2, and A3). **D** Venn Diagram and Fisher’s exact test depicting significant overlap between the hypermethylated transcripts following chronic morphine treatment or *Alkbh5* knock-down in primary cortical cultures with > 2 fold change. **E** Pie chart of the expression of the top 25th percentile of cell-specific marker genes in primary cortical cultures. Analysis of expression levels of 150 cell-type specific markers for astrocytes, microglia, or neurons was performed to determine the presence of multiple cell types in primary cortical cultures. Pie graph depicts the proportion of astrocyte-enriched, microglia-enriched, or neuron-enriched genes that are most highly expressed out of the 150 cell-specific markers. **F**–**I** Gene Ontology analysis for biological process for differentially expressed genes at > 2 fold change: **F** morphine m6A hypermethylated transcripts, **G** morphine m6A hypomethylated transcripts, **H** *Alkbh5* knock-down m6A hypermethylated transcripts, **I** common m6A hypermethylated transcripts between morphine treatment and *Alkbh5* knock-down

In analyzing the mRNA and m6A methylation datasets, we evaluated the outcome of imposing a more stringent cutoff criteria of > 2.0 fold change (Fig. 6A–C). Importantly, significant overlap was observed between the m6A hypermethylated transcripts resulting from morphine or *Alkbh5* knock-down when imposing the > 2.0 fold change cutoff, with ~ 33% of morphine-induced m6A differentially hypermethylated transcripts commonly regulated by *Alkbh5* knock-down (Fisher’s exact test: *p* < .0001; Fig. 6D). Seventeen hypermethylated transcripts that were common to the two treatments at > 2.0 FC cutoff criteria can be found in Supplemental Figure 2. Moreover, the Gene Ontology terms for Biological Processes associated with m6A methylated transcripts showed significant overlap between the 1.5 and 2.0 fold change cutoff criteria for morphine m6A hypermethylated, morphine m6A hypomethylated and *Alkbh5* knock-down m6A hypermethylated (Supplemental Figure 3). When comparing the impact of m6A differential methylation from either morphine or *Alkbh5* knock-down on mRNA expression at > 2 fold change, we also observed significant overlap in the regulation of transcripts that were differentially expressed (Fisher’s exact tests, *p* < 0.0001 for morphine vs. vehicle or *Alkbh5* knock-down vs. scrambled; Supplemental Figure 4). These results indicate that the top pathways or groups of genes regulated in our experimental design target the same pathways for both stringency criteria.

The pathway analysis and Gene Ontology indicated enrichment of pathways for immune-related terms in m6A differentially methylated gene. Because immune-related genes are typically expressed at higher levels in non-neuronal cells such as astrocytes and microglia, we analyzed the expression of cell-enriched markers in the mRNA data obtained from the microarray. Using previously published lists of cell-enriched markers from a tool called BRETIGEA that seeks to perform “deconvolution” of gene expression datasets involving bulk-tissue samples, we calculated the average expression value for all samples of cortical cultures for genes known to be enriched in astrocytes, microglia, or neurons [39]. The results can be found in Supplemental Excel Table SE7. We conclude the genes with the top 25th percentile of expression amongst the cell-specific markers were composed of genes from a mixture of all three cell types, with the majority (~ 42% from neurons) (Fig. 6E). This data indicates that astrocyte and microglia were likely present in the cortical cultures and may have contributed to the gene expression findings presented in this study.

### Morphine and *Alkbh5* Knock-Down Induce Differential Methylation of Non-coding RNAs

**Fig. 7:**
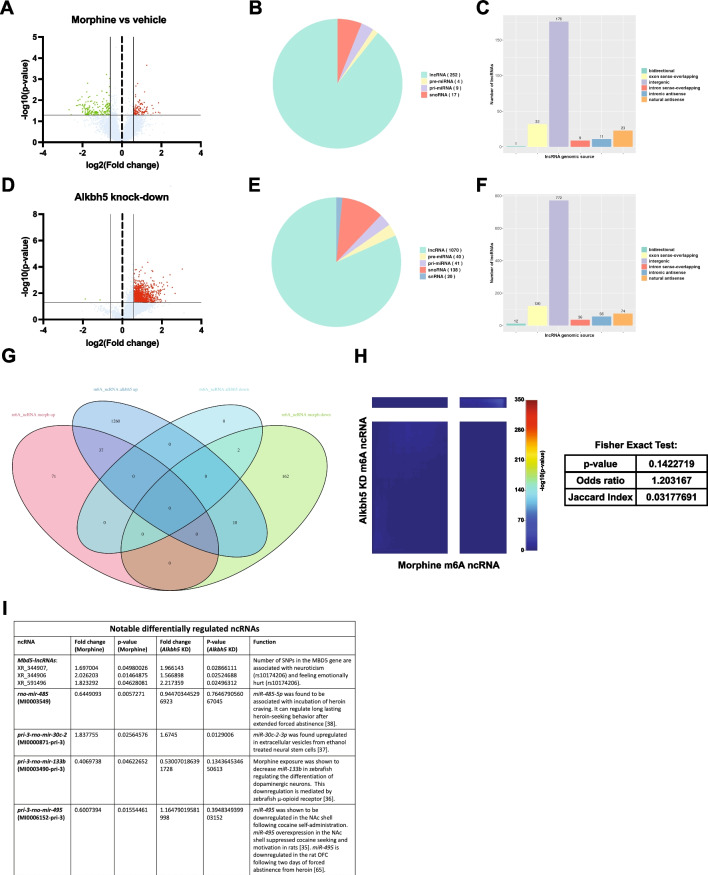
Chronic morphine treatment and *Alkbh5* knock-down result in differential m6A methylation of non-coding RNAs (ncRNAs). **A** Volcano plot depicting differential m6A methylation of ncRNA transcripts in primary cortical cultures following chronic morphine treatment. **B** Type of differentially methylated ncRNAs following morphine treatment. **C** Genomic origin of the differentially methylated long non-coding RNAs (lncRNAs) following morphine treatment. **D** Volcano plot depicting differential m6A methylation of ncRNA transcripts in primary cortical cultures following *Alkbh5* knock-down. **E** Type of differentially methylated ncRNA following *Alkbh5* knock-down. **F** Genomic origin of the differentially methylated ncRNAs following *Alkbh5* knock-down. **G** Venn diagram of the overlap between hyper- and hypomethylated ncRNAs following chronic morphine treatment and *Alkbh5* knock-down. (H) RRHO plot and Fisher exact test showing lack of coordinated gene expression between m6A epitranscriptomic profile of ncRNAs following morphine treatment and *Alkbh5* knock-down. **I** Summary table with selected differentially methylated ncRNAs following either morphine treatment or Alkbh5 knock-down that were previously shown to have a role in substance use and other psychiatric conditions

In addition to mRNA m6A methylation, the epitranscriptomic array quantifies m6A methylation for noncoding (nc) RNAs, including microRNA and long ncRNA (lncRNA). We next examined how both morphine treatment and *Alkbh5* knock-down affect the m6A epitranscriptome of these other RNA species in primary cortical cultures. We observed differential m6A methylation for 282 ncRNA transcripts following morphine, of which 89% were lncRNAs. Very few snoRNAs (0.6%) and pri-microRNAs (0.03%) or pre-microRNAs (0.014%) were impacted by morphine at the level of m6a methylation (Fig. 7A, B and Supplemental Excel Table SE8). The majority of the impacted lncRNAs were intergenic, with a few exon-sense overlapping, antisense or intron sense-overlapping (Fig. 7C). *Alkbh5* knock-down led to differential m6A methylation of 1309 ncRNAs: 1070 lncRNAs (81.7%), 138 snoRNAs (10.5%), 20 snRNAs (1.5%), 41 pri-microRNAs (3.1%), and 40 pre-microRNAs (3.1%) (Fig. 7D, E and Supplemental Excel Table SE9). Most differentially m6A-methylated lncRNAs were also intergenic (Fig. 7F). Overlap between the differentially m6A-methylated ncRNAs following either morphine treatment or *Alkbh5* knock-down revealed that 13.8% of morphine-driven m6A changes to ncRNA were also regulated in the same direction by *Alkbh5* knock-down (Fig. 7G). However, the overlap between the two datasets for m6a methylation of ncRNAs was not significant (Fisher’s exact test: *p*-value = 0.142) (Fig. 7H and Supplemental Excel Table SE10). Morphine treatment led to differential m6A methylation of a number of ncRNAs that were previously associated with drug seeking, drug induced neuroadaptations, or personality traits associated with drug use: *Mbd5-lncRNA*, *rno-mir-485*, *pri-3-rno-mir-30c-2*, *pri-3-rno-mir-133b*, and *pri-3-rno-mir-495* (Fig. 7I) [40–43].

**Fig. 8:**
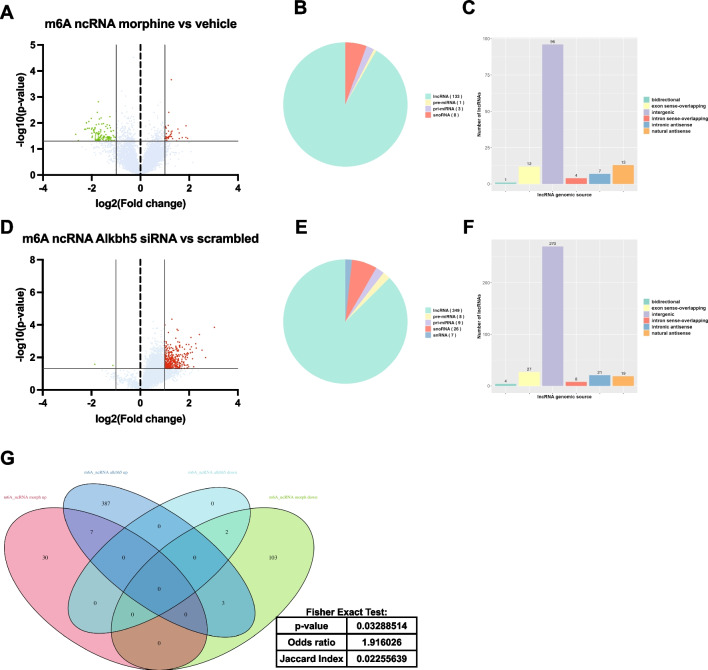
Chronic morphine treatment and *Alkbh5* knock-down result in differential m6A methylation of non-coding RNAs (ncRNAs) using a cutoff criteria of > 2.0 fold change. **A** Volcano plot depicting differential m6A methylation of ncRNA transcripts in primary cortical cultures following chronic morphine treatment using a fold change criteria of > 2.0. **B** Type of differentially methylated ncRNAs following morphine treatment observed in **A**. **C** Genomic origin of the differentially methylated long non-coding RNAs (lncRNAs) following morphine treatment. **D** Volcano plot depicting differential m6A methylation of ncRNA transcripts in primary cortical cultures following *Alkbh5* knock-down using a fold change criteria of > 2.0. **E** Type of differentially methylated ncRNA following *Alkbh5* knock-down observed in **D**. **F** Genomic origin of the differentially methylated ncRNAs following *Alkbh5* knock-down. **G** Venn diagram and Fisher’s exact test of the overlap between hyper- and hypomethylated ncRNAs following chronic morphine treatment and *Alkbh5* knock-down using a fold change criteria of > 2.0

We also performed a more stringent analysis of the epitranscriptomic dataset of ncRNAs using the > 2.0 FC criteria. We observed differential m6A methylation of 145 transcripts following morphine and 399 following *Alkbh5* knock-down, with the majority of the regulation occurring in lncRNA transcripts that were intergenic (Fig. 8A–F). Notably, the overlap between the two datasets for m6a methylation of ncRNAs was significant (Fisher’s exact test: *p*-value = 0.033) (Fig. 8G), with 8.3% of morphine-regulated m6A differentially methylated transcripts shared by *Alkbh5* knock-down.

### Gene-Drug Interaction Analysis and Comparison with Human OUD Datasets

We evaluated predicted drug interactions for the differentially m6A methylated genes following chronic morphine treatment and *Alkbh5* knock-down and identified 777 interactions for morphine and 5309 interactions for *Alkbh5* knock-down (Supplemental Excel Tables SE11, 12). In addition, we observed interactions with 13 opioids (Table 1). We found one transcript that was differentially methylated in both morphine and *Alkbh5* knock-down with a predicted interaction with fentanyl: xanthine dehydrogenase (*Xdh*). Among the morphine differentially m6A-methylated transcripts, we found interactions with opioids for *Xdh*, mitogen-activated protein kinase 14 (*Mapk14*), and nuclear receptor subfamily 1 group I member 3 (*Nr1I3*). Among the differentially methylated transcripts following *Alkbh5* knock-down, we found interactions with opioids for cholecystokinin B receptor (*Cckbr*), purinergic receptor P2X 7 (*P2rx7*), brain derived neurotrophic factor (*Bdnf*), acetylcholinesterase (*Ache*), RUNX family transcription factor 1 (*Runx1*), lysophosphatidic acid receptor 2 (*Lpar2*), 5-hydroxytryptamine receptor 3B (*Htr3b*), Fc gamma receptor IIIa (*Fcgr3a*), dopamine receptor D2 (*Drd2*), dopamine receptor D4 (*Drd4*), glutamate ionotropic receptor NMDA type subunit 1 (*Grin1*), microtubule associated protein tau (*Mapt*), *Nr1I3*, mannose phosphate isomerase (*Mpi*), post-GPI attachment to proteins 6 (*Pgap6*), ATP-binding cassette subfamily C member 3 (*Abcc3*), myeloperoxidase (*Mpo*), *Xdh*, aldehyde dehydrogenase 2 family member (*Aldh2*), calcium voltage-gated channel subunit alpha1 E (*Cacna1e*), and C-C motif chemokine ligand 11 (*Ccl11*) (Table 1).

**Table 1:**
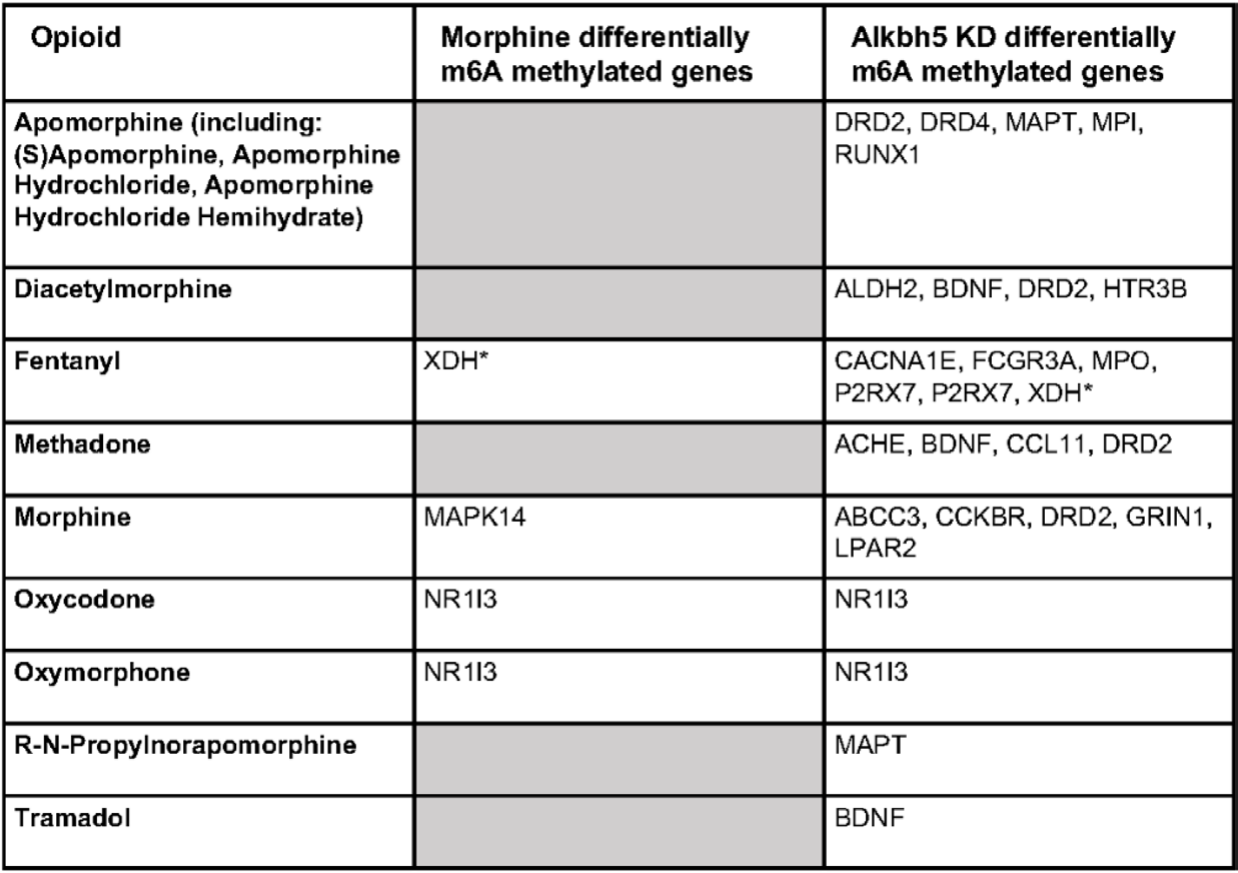
Summary table of the drug interaction analysis for opioids for differentially m6A-methylated genes driven by morphine treatment or *Alkbh5* knock-down

**Fig. 9:**
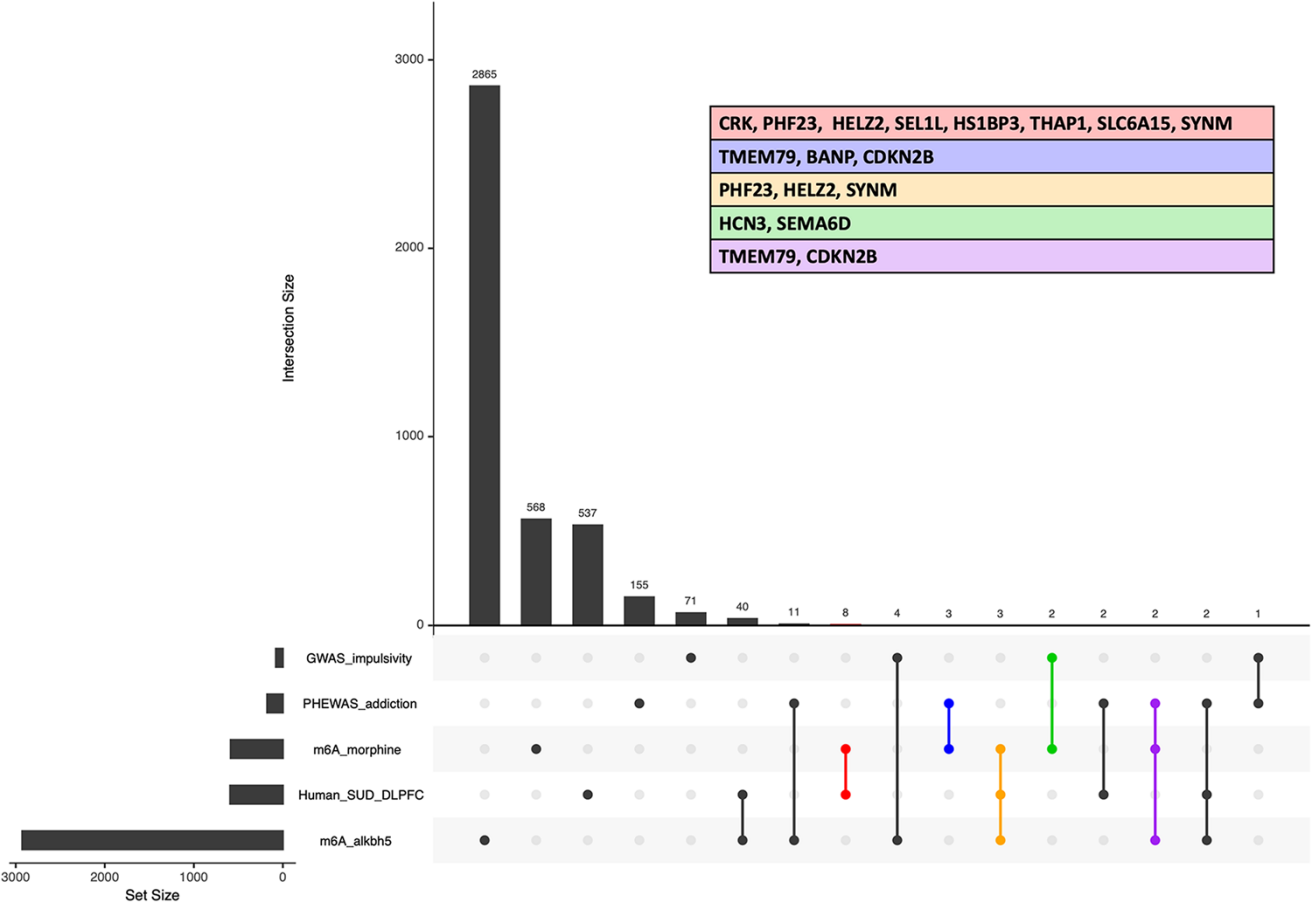
Integration of differentially m6A-methylated transcripts following morphine or *Alkbh5* knock down with human datasets. UpSet plot visualization of the overlap between differentially m6A-methylated transcripts following morphine treatment or *Alkbh5* knock-down (> 1.5 FC), human OUD transcriptomics [44], impulsivity trait GWAS data [45], and PheWAS [46]. The *y*-axis–intersection size represents the number of genes found in overlapping datasets. The color-coded table contains common genes between the datasets

To explore our work in the context of previously published human studies, we looked at the overlap between differentially m6A-methylated transcripts following morphine treatment or *Alkbh5* knock-down and published RNA sequencing (RNAseq) datasets from human subjects with OUD [44], a genome-wide association study (GWAS) of impulsive personality traits [45], and a phenome-wide association study (PheWAS) for substance use disorders (SUDs) [46]. The overlap between datasets is present on the UpSet plot shown in Fig. 9. We observed a small subset of differentially m6A-methylated transcripts that overlapped with the human transcriptomics from the dorsolateral prefrontal cortex (DLPFC) of OUD subjects (8 transcripts), PheWAS for addiction (3 transcripts), and GWAS for impulsivity (2 transcripts). Three transcripts overlapped between m6A-morphine, m6A-*Alkbh5* knock-down, and the human OUD dataset: PHD finger protein 23 (PHF23), helicase with zinc finger 2 (HELZ2), and synemin (SYNM). Two transcripts were common between m6A-morphine, m6A-*Alkbh5* knock-down, and the PheWAS dataset: transmembrane protein 79 (TMEM79) and cyclin dependent kinase inhibitor 2B (CDKN2B) (Fig. 9). Table 2 summarizes the data for transcripts overlapping between our m6A datasets and previously published studies. This data points to potentially important genes for OUD that may be regulated by opioids at the level of m6A RNA methylation.

**Table 2:**
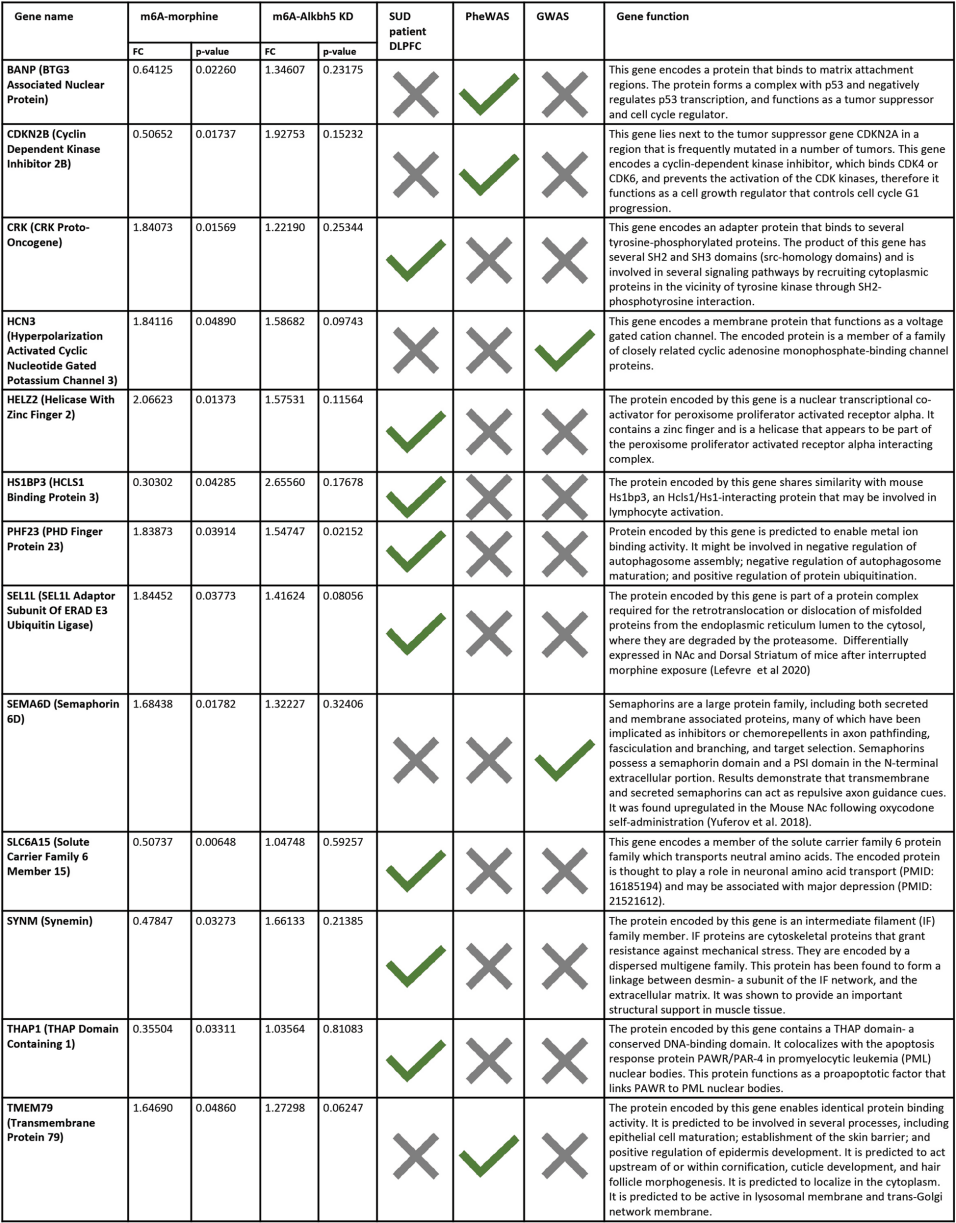
Summary table of differentially m6A-methylated transcripts that overlap with previously published human OUD-related datasets. *SUD patient DLPFC*, RNAseq detected transcriptional alterations in the DLPFC of human opioid use patients [44]; *PheWAS*, phenome wide association study filtered for “Substance addiction and disorders” [46]; *GWAS*, genome wide association study of impulsive personality traits [45]

## Discussion

This study represents an essential first investigation into opioid-induced m6A epitranscriptomic changes in the nervous system. We have established a comprehensive atlas of m6A epitranscriptomic neuroadaptations induced by morphine and siRNA knock-down of the m6A eraser *Alkbh5*. We have demonstrated a significant overlap between the differentially m6A-methylated transcripts following morphine treatment and *Alkbh5* knock-down. Morphine treatment and *Alkbh5* knock-down cause concordant differential methylation at the mRNA level, suggesting that morphine-induced m6A changes could be, in part, caused by the observed downregulation of *Alkbh5*. Finally, the comparison of our m6A microarray results with previously published RNAseq data from the DLPFC of human OUD subjects, GWAS, and PheWAS datasets identified potential key molecular targets that are conserved in their regulation by opioids.

Recent studies have associated aberrant m6A changes with a number of psychiatric disorders of the reward circuitry [24, 25, 47, 48]. However, thus far, opioid-induced m6A epitranscriptomic neuroadaptations have not been described. Opioids are well known to cause transcriptomic and epigenetic changes [4, 27, 43, 44, 49–52]. Such opioid-induced transcriptomic neuroadaptations, including changes in mRNA levels, ncRNA levels, and changes in alternative splicing, can be, in part, explained by epigenetic shifts. However, we still do not fully understand the mechanisms by which they occur, and it is plausible that m6A modifications may contribute to opioid-induced transcriptomic dysregulation. It was well established that m6A modifications regulate mRNA metabolism [19], splicing [53], and cellular localization [54] which places them in a unique position as a potential culprit regulating opioid-induced neuroadaptations.

Morphine-induced m6A epitranscriptomic changes affected transcripts associated with inflammation, infection, and actin cytoskeleton. Prior studies have shown that opioids such as heroin or morphine can affect immunity-related pathways [55, 56]. A number of studies have reported an immunosuppressive effect of opioids through studies in the spleen or the peripheral blood looking at decreased natural killer (NK) cell activation, reduced responses to mitogens, depression of antibody production or suppression of phagocytosis [55]. In addition, there is a rich body of knowledge supporting the notion that opioid use and opioid exposure lead to the development of neuroinflammation [56]. Both rodent in vivo and in vitro studies have reported upregulation of proinflammatory cytokines in microglia cell cultures and the nervous system, such as IL1-β, IL-6, and TNF-⍺, in response to opioid exposure [57–63]. Interestingly, among the hyper and hypomethylated genes following chronic morphine treatment, we observed an enrichment for genes associated with inflammation and infection-related terms, such as *Staphylococcus aureus* infection, HIV-1 infection, and Epstein-Barr virus infection. Another previously described opioid-induced neuroadaptation involves changes in the actin cytoskeleton, a cellular component that, in neurons, is important for synapse formation, long-term potentiation (LTP), and the growth and morphology of dendritic spines [64–67]. A number of studies in the recent years have implicated reorganization of the actin cytoskeleton in possibly mediating opioid-induced synaptic plasticity changes [64, 65, 68, 69]. Indeed, we observed hypermethylation of genes important for the regulation and maintenance of the actin cytoskeleton, such as *Tmod2*, *Fmn1*, *Actn4*, and *Myo1e*. In addition, synaptic mapping using SynGO and the recently published synaptosome proteomic dataset revealed differential methylation of transcripts mapped to the synapse. It remains to be determined what are the functional synaptic implications of these differential m6A methylation events. This data suggests that some of the neuroinflammation and actin cytoskeleton-related neuroadaptations in response to morphine could be mediated by changes in the m6A epitranscriptome.

In our study, we report that morphine exposure not only affects the m6A methylation state of mRNAs, but also it can impact non-coding RNA species such as lncRNAs, miRNA, and snoRNAs. We demonstrated that chronic morphine treatment leads to differential methylation of ncRNA transcripts that were previously associated with drug seeking, exposure, or the reward system: *rno-mir-485*, *pri-3-rno-mir-133b*, *pri-3-rno-mir-495*, and *pri-3-rno-mir-30c-2*. We have previously shown that *miR-485-5p* is downregulated within the rat orbitofrontal cortex following 21 days of forced abstinence from heroin and can regulate heroin seeking behavior following forced abstinence [43]. In a recent study, *miR-495* is downregulated in the orbitofrontal cortex of rats following 2 days of acute forced abstinence from heroin [70]. Morphine-induced downregulation of *miR-133b* is believed to be mediated through the μ opioid receptor [41]. *miR-495* was downregulated in the mouse nucleus accumbens (NAc) shell following cocaine self-administration and *miR-495* overexpression within the NAc shell can suppress cocaine seeking and motivation [40]. Lastly, *miR-30c-2-3p* was upregulated in extracellular vesicles from ethanol treated neural stem cells [42]. Interestingly, the morphine hypomethylated miRNAs such as *rno-mir-485*, *pri-3-rno-mir-133b*, and *pri-3-rno-mir-495* were shown to be downregulated in the aforementioned studies involving drugs, while the hypermethylated miRNA, *pri-3-rno-mir-30c-2*, was shown to be upregulated following ethanol exposure. Increased, m6A methylation of transcripts tends to have a stabilizing effects and therefore corresponding expression changes in prior literature strengthen the connection between these ncRNAs and morphine-induced neuroadaptations.

We hypothesized that differential regulation of m6A-related enzymes would lead to differential m6A epitranscriptomic states in primary cortical cultures. We observed the downregulation of the m6A erasers *Fto* and *Alkbh5* following chronic morphine treatment and theorized that the m6A epitranscriptomic changes that were observed may be due to dysregulation of such enzymes. We would expect that downregulation of m6A erasers should lead to m6A hypermethylation of transcripts. There already is a high volume of research on the biological function of *Fto* in health and disease, including in the context of the reward system and drugs of abuse [20, 71–74]. However, *Alkbh5* is significantly understudied in this regard. As expected, *Alkbh5* knock-down led to selective m6A hypermethylation events. The affected transcripts were enriched for immunity-related pathways as well as for cell adhesion. Our bioinformatic analysis of the m6A epitranscriptomic changes induced either by morphine or *Alkbh5* knock-down showed significant overlap between the significantly regulated mRNA transcripts, as well as concordance in regard to the two m6A epitranscriptomic profiles. The shared hypermethylated mRNA transcripts are involved in immunity, cell adhesion, regulation of the actin cytoskeleton, and MAPK signaling, suggesting that these morphine-induced neuroadaptations may be regulated, in part, through *Alkbh5* downregulation. On the other hand, we showed lack of concordance and no significant overlap between the m6A methylation changes of ncRNA transcripts, suggesting that morphine-induced m6A methylation shifts of ncRNAs are not connected to *Alkbh5* downregulation. It is worth noting that *Alkbh5* knock-down induced a significantly higher number of hypermethylated transcripts than morphine exposure. This result could be due to a number of reasons. First, more variability was observed in the vehicle-treated samples that morphine-treated samples were compared to than in the samples treated with siRNAs. In addition, *Alkbh5* knock-down is a very targeted approach where we evaluate the effects of downregulation of a single m6A enzyme that likely has many putative transcriptional substrates. In contrast, morphine, induces signaling pathways downstream of opioid receptor signaling, but may have more transient effects during the timeframe that we have examined. One possibility is that morphine does impact both m6A erasers and writers and the dynamic balance of epitranscriptomic regulation that occurs in response to morphine is substrate-specific. While we observed differential regulation of the m6A erasers, *Alkbh5* and *Fto*, that presumably led to observed hypermethylation events; however, the observed trend towards significance for *Mettl3* suggests a possible compensatory mechanism potentially counteracting the aforementioned eraser downregulation.

In the recent years, human substance misuse and addiction-related resources in the form of GWAS, PheWas, and RNAseq data from human SUD patients were developed, and therefore, looking at similarities between preclinical or in vitro data and human data is very important to gauge the translatability of these studies and to inform future ones. We therefore examined how our rat primary cortical culture m6A epitranscriptomic microarray data relates to these human SUD-relevant resources. Among the 568 differentially m6A-methylated mRNA transcripts following morphine treatment, a total of 13 transcripts were shared with either the RNAseq data from the DLPFC of OUD human subjects (8 transcripts), GWAS data for impulsivity (2 transcripts), or PheWAS data for addiction (3 transcripts). Two of the overlapping genes, BANP and CDKN2B, are important cell cycle regulators [75, 76]. SYNM encodes an intermediate filament that in neurons binds to neurofilaments impacting cell shape and was shown to regulate a number of signaling pathways binding to protein kinase A and protein phosphatase type 2A [77]. HCN3 is a membrane protein that acts as voltage gated cation channel [78]. In addition, some of these gene candidates, such as SEL1L and PHF23, are involved in protein metabolism through participation in protein trafficking of misfolded proteins or ubiquitination [79, 80]. Interestingly, *Sel1l* was found to be upregulated in the NAc and dorsal stratum of mice after interrupted morphine exposure [81]. Thus, these commonly occurring transcripts could serve as promising targets for future mechanistic studies into the consequences of opioid-induced neuroadaptations.

Our study utilized rat primary cortical cultures to perform microarray-based profiling of m6A modifications following morphine exposure, then compared the generated m6A dataset with previously published human studies, but is not without limitations. We acknowledge that the primary cortical cultures were prepared with methodology to yield enrichment for neurons, but a limitation of the study is potential co-culturing of a small portion of microglial and astrocytic cells. This may contribute to the enrichment of pathways for immune-related terms and genes that are typically expressed at higher levels in microglia, such as C1qa and C1qb. Therefore, we are not able to delineate the cell-type specific regulatory mechanisms induced by morphine and *Alkbh5* knock-down in our study. In addition, we only utilized cortical cultures, but evaluating whether observed epitranscriptomic changes are conserved between brain regions is also a necessary avenue to investigate. While we implemented an analysis to “deconvolute” the cell types present in our cortical cultures, we only examined the expression of microglial, neuronal and astrocytic marker genes in our study. Classical deconvolution methods also examine OPGs, oligodendrocytes, and endothelial cells and such approaches will be helpful in future studies of bulk tissue gene expression analyses to attempt to speak to the cell-types present in cortical cultures. Moreover, future studies should address these limitations by employing methodologies to characterize cell-type specific epitranscriptomic responses, such as single-cell RNA sequencing or flow cytometry combined with transcriptomics, as well as evaluate these mechanisms in cells from other brain regions relevant to OUD pathology, including the nucleus accumbens, ventral tegmental area, and dorsal striatum. Second, we have shown that morphine exposure leads to a downregulation of *Fto* and *Alkbh5*, and we reported a trend towards downregulation of *Mettl3*. However, in our study, we focused on evaluating the extent of *Alkbh5* mediating the m6A epitranscriptomic changes induced by morphine. Future studies looking into the role of *Fto*, *Mettl3*, and possibly other m6A relevant enzymes in mediating the morphine driven m6A methylation changes are imperative for understanding opioid-induced epitranscriptomic neuroadaptations. Future studies should further examine the extent to which overexpression of *Alkbh5* may reverse morphine-induced epitranscriptomic profiles. Another limitation of the study is the use of unadjusted *p*-values to determine statistical significance for the microarray data, which may result in some false positives. Analysis of the microarray data using adjusted *p*-value did not yield any transcripts with statistical significance, which is likely due to limitations in sample size. However, we have sought to overcome this limitation and report the most meaningful morphine-induced epitranscriptomic consequences by overlapping the morphine-exposed samples to those that had knock-down of *Alkbh5*. By comparing two independent experiments, we have identified epitranscriptomic changes that are commonly altered by each experimental condition, which may reduce the incidence of false positives. Finally, the comparison of the rat m6A epitranscriptomic data with human studies has shown a very limited overlap. It is crucial to note that this comparison involves not only contrasting alterations between human and rat data—two species known for their distinct responses—but also juxtaposing RNA m6A methylation profiles with DNA-level SNP association data and total RNA data obtained from post-mortem individuals.

In summary, the present study identified differential m6A methylation of both ncRNA and mRNA transcripts in primary cortical cultures in response to morphine. Our results suggest that m6A marks may play an important role in mediating opioid-induced neuroadaptations, such as changes in actin cytoskeleton, cell adhesion, and neuroinflammation. We have identified *Alkbh5* as a potential facilitator of a subset of these epitranscriptomic changes. Finally, by comparing our results with human OUD data, we have identified a number of potential opioid-regulated genes that could inform future studies.

## Supplementary Information

ESM 1 (XLSX 3623 kb)

ESM 2 (PDF 1007 kb)
