# Supplementary material for: Morphine-Driven m6A Epitranscriptomic Neuroadaptations in Primary Cortical Cultures
Source: Mol Neurobiol. 2024 May 23;61(12):10684–704. doi: 10.1007/s12035-024-04219-z (PMC11584444; doi:10.1007/s12035-024-04219-z)
Supplement: Supplementary file 2 — (PDF 1007 kb) [file 12035_2024_4219_MOESM2_ESM.pdf]

Supplementary Materials for “Morphine-driven m6A epitranscriptomic neuroadaptations in primary cortical cultures.” by Dabrowski et al.

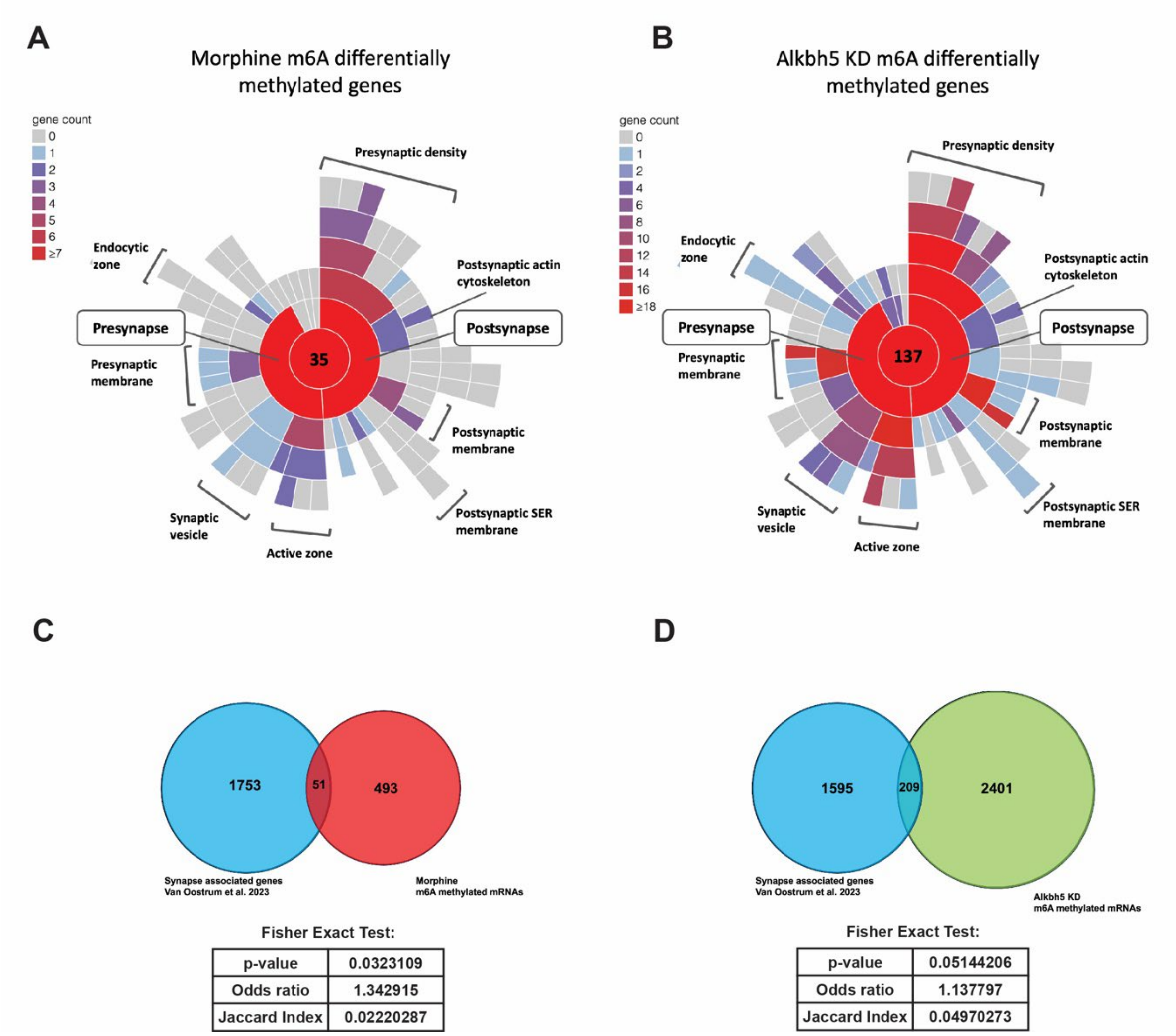

Supplementary Figure 1. Synaptic mapping of differentially m6A methylated transcripts. (A, B) SynGO-generated sunburst plots reveal different synaptic locations for differentially m6A-methylated genes following chronic morphine treatment (A) or *Alkbh5* knock-down (B). Sunburst plots depicting the synaptic locations that start with the synapse in the center, pre- and post-synaptic locations within the first ring, and child terms in the subsequent rings. The number of genes for each location is depicted by the

color scheme in the legend. (C-D) Venn diagrams depicting the overlap between m6A differentially methylated transcripts and genes associated with the synapse by fluorescent-activated synaptosome sorting followed by proteomic analysis by van Oostrum et al 2023. Summary tables depict Fisher exact test used to evaluate the significance of the overlap.

m6A hypermethylated common transcripts between morphine treatment and *Alkbh5* knock-down				
**Gene name**	**Fold change (Morphine)**	**p-value (Morphine)**	**Fold change (*Alkbh5* KD)**	**p-value (*Alkbh5* KD)**
**Kng1**	3.22970	0.02270	4.05831	0.02024
**Zyg11a**	3.21272	0.00401	3.33527	0.00206
**RGD1561145**	3.03173	0.00486	2.19992	0.00865
**AABR07039736.1**	2.69060	0.00714	4.47583	0.00098
**Tmem132c**	2.66955	0.03856	2.93721	0.03530
**Olr91**	2.59321	0.03905	3.83017	0.01062
**C1qb**	2.35212	0.03093	2.53658	0.00452
**Adgrf5**	2.32297	0.04339	5.40317	0.00084
**Il18rap**	2.28899	0.02122	3.11417	0.00194
**Rnf151**	2.26705	0.03456	2.92718	0.00706
**AABR07062183.1**	2.18448	0.03735	3.01130	0.00509
**RGD1305807**	2.16278	0.04312	2.32559	0.01594
**Foxa1**	2.13921	0.03715	6.55996	0.00285
**Deup1**	2.13842	0.00285	4.35168	0.03197
**Klra5**	2.12637	0.03114	2.46819	0.04365
**Trem3**	2.10775	0.01191	2.12955	0.04548
**AABR07060352.2**	2.10571	0.02484	4.09069	0.02169

Supplementary Figure 2. m6A hypermethylated common transcripts between morphine treatment and *Alkbh5* knockdown following implementation of >2 fold change criteria. A table of the 17 transcripts that are commonly hypermethylated by morphine and *Alkbh5* knock-down with fold change >2.

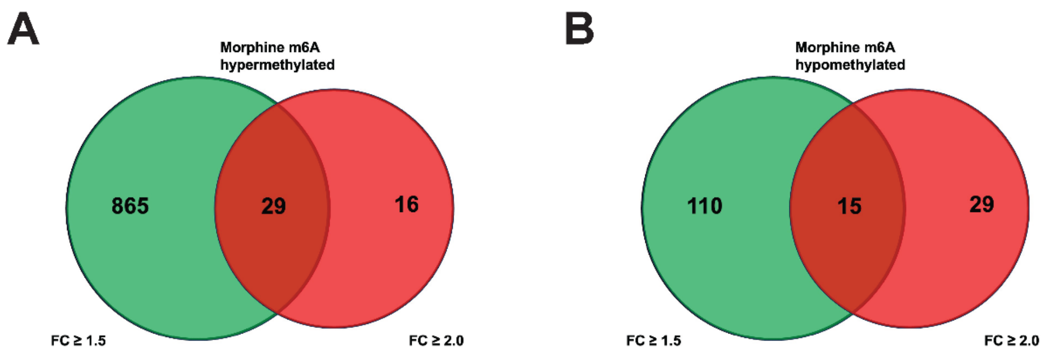

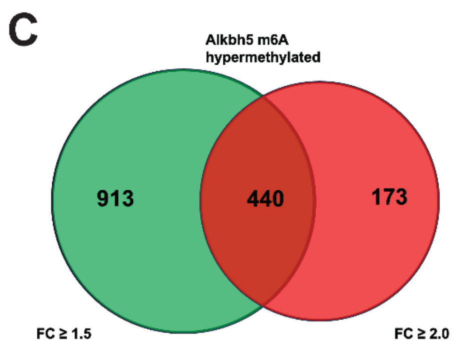

D

Fisher Exact Test p-values:

Morphine hyper-	1.398515e-38
Morphine hypo-	4.142548e-28
Alkbh5 hyper-	2.29049e-264

Supplementary Figure 3: Significant overlap of Gene Ontology terms for Biological Processes for m6A methylated transcripts between morphine treatment and *Alkbh5* knockdown is maintained following implementation of >2 fold change criteria. (A-C) Venn Diagrams comparing the overlap of the Gene Ontology terms for Biological Process of m6A methylated transcripts observed with fold change cutoff criteria of 1.5 fold versus 2 fold for morphine (A, B) or *Alkbh5* knock-down (C). (D) Values of Fisher exact test comparing the overlap between the common gene ontology terms for the 1.5 and 2.0 fold change criteria.

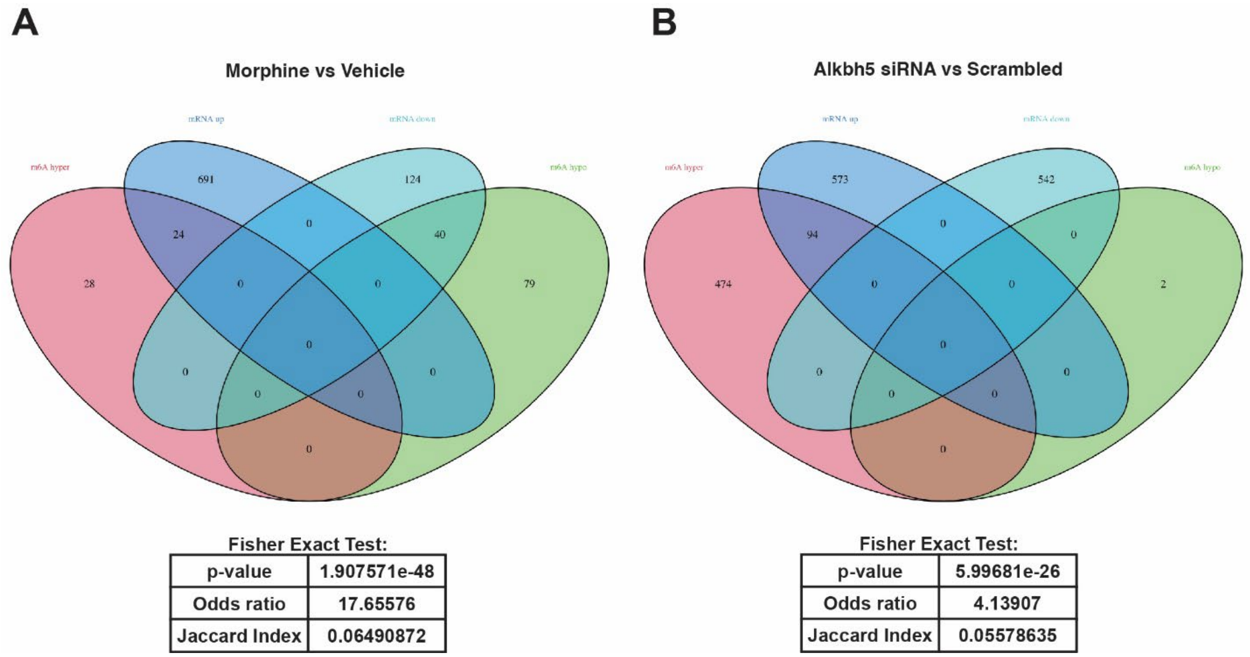

Supplementary Figure 4. Differential m6A methylation is accompanied by altered RNA expression after chronic morphine treatment or *Alkbh5* knock-down that is also observed with a higher fold change cutoff of >2.0.

Venn diagrams depicting overlap between m6A hypermethylated (m6A hyper), m6A hypomethylated (m6A hypo), mRNA upregulated (mRNA up), and mRNA downregulated (mRNA down) transcripts following morphine treatment (A) or *Alkbh5* knock-down (B) for mRNA transcript and m6A methylation that was >2.0 fold change.

mRNA gene expression assays		
**Target**	**Oligo type**	**Sequence**
ActB	Probe	5'-/ 56-FAM/TCCTGGGT A/Z EN/TGGAA TCCTGTGGC/31ABkFQ/-3'
	Primer 1	5'-TCACTATCGGCAATGAGCG-3'
	Primer 2	5'-GGCATAGAGGTCTTTACGGATG-3'
Alkbh5	Probe	5'-/56-FAM/CAGCAGCAT /ZEN/ ACCCACTGAGCACA/31ABkFQ/-3'
	Primer 1	5'-AGTTCCAGTTCAAGCCCATC-3'
	Primer 2	5'-GCGTTCTTTGATGTCCTGAG-3'
Fto	Probe	5'-/ 56-FAM/CAGAACTGC/Z EN/ AGGCTCGAAGGACT /31ABkFQ/-3'
	Primer 1	5'-TCTCAATGACTCGGACAATGG-3'
	Primer 2	5'-TGAAACCAGAACTGCCTCAG-3'
Gapdh	Probe	5'-/ 56-FAM/TCTGCCTTC/Z EN/TT AGAGCCA TTCAGCG /31ABkFQ/-3'
	Primer 1	5'-GCATCAGTGTCATGGTATCTCG-3'
	Primer 2	5'-CACAAACATCATCACCCACAC-3'
Mettl3	Probe	5'-/56-FAM/ ACTCTGGGC/ZEN/ ACTTGGACTT AAGGAATC/31ABkFQ/-3'
	Primer 1	5'-T AAGAAACAACTGGACTCGCT-3'
	Primer 2	5'-GCTATCACTACGGAAGGTTGG-3'
Ythdc1	Probe	5'-/56-FAM/TGATGATTT /ZEN/CCTTCGCCGCACACA/31ABkFQ/-3'
	Primer 1	5'-CCACATGAAGCAAGATACAGAGA-3'
	Primer 2	5'-TCT ACTTCTCCGACCACTGAC-3'

Supplementary Table 1. Primers and probes used to measure mRNA expression.

siRNA pools		
**Target**	**#**	**Sequence**
Alkbh5	1	5'-CUGAGAACUAUUGGCGCAA-3'
	2	5'-GAUUAGAUGCACCACGAUU-3'
	3	5'-CCAGAGACCCUGCGCUGAA-3'
	4	5'-CCAAAAGGUCCCACCGCAA-3'
Scrambled control	1	5'-UGGUUUACAUGUCGACUAA-3'
	2	5'-UGGUUUACAUGUUGUGUGA-3'
	3	5'-UGGUUUACAUGUUUUCUGA-3'
	4	5'-UGGUUUACAUGUUUUCCUA-3'

Supplementary Table 2. siRNA sequences used for knock-down experiments.

Supplementary Excel Tables:

SE1 Significantly differentially m6A-methylated mRNA transcripts following morphine treatment

SE2 Significantly differentially m6A-methylated mRNA transcripts following Alkbh5 knock-down

SE3 SynGO analysis of differentially methylated genes following morphine treatment

SE4 SynGO analysis of differentially methylated genes following Alkbh5 knock-down

SE5 Commonly m6A hypermethylated mRNA transcripts between morphine treatment and Alkbh5 knock-down

SE6 RRHO data for Morphine and Alkbh5 knock-down - m6A change of mRNA and total mRNA change

SE7 Average mRNA expression of cell-enriched markers in primary neuronal cultures

SE8 Significantly differentially m6A-methylated ncRNA transcripts following morphine treatment

SE9 Significantly differentially m6A-methylated ncRNA transcripts following Alkbh5 knock-down

SE10 RRHO data for ncRNA analysis

SE11 Drug-Gene Interaction analysis for differentially methylated genes following morphine treatment

SE12 Drug-Gene Interaction analysis for differentially methylated genes following Alkbh5 knock-down
